# Time-restricted eating with calorie restriction on weight loss and cardiometabolic risk: a systematic review and meta-analysis

**DOI:** 10.1038/s41430-023-01311-w

**Published:** 2023-07-24

**Authors:** Jing-Chao Sun, Zhen-Tao Tan, Chao-Jie He, Hui-Lin Hu, Chang-Lin Zhai, Gang Qian

**Affiliations:** 1 Department of Cardiology, The Affiliated Hospital of Jiaxing University, Jiaxing, Zhejiang 314000 China; 2 Jiaxing University Master Degree Cultivation Base, Zhejiang Chinese Medical University, Jiaxing, Zhejiang 314000 China

**Keywords:** Obesity, Weight management, Nutrition, Lifestyle modification

## Abstract

The effect of time-restricted eating (TRE) has been summarized in previous studies, but its benefits in combination with calorie restriction (CR) still need to be determined. The present meta-analysis aimed to evaluate the efficacy of TRE with CR on weight loss and cardiometabolic risk. PubMed, Embase, Cochrane Library, and gray literature databases were searched from inception to October 18, 2022, for potential randomized controlled trial (RCT) studies based on predefined inclusion and exclusion criteria. Body weight and other cardiometabolic risk factors were described as weighted mean difference (WMD) with a 95% confidence interval (CI). Eight RCTs involving 579 participants were enrolled in the present analysis. The pooled results showed that TRE with CR reduced the body weight, fat mass, and waist circumference significantly (WMD: −1.40, 95% CI: −1.81 to −1.00, and I^2^: 0%; WMD: −0.73, 95% CI: −1.39 to −0.07, and I^2^: 0%; WMD: −1.87, 95% CI: −3.47 to −0.26, and I^2^: 67.25%, respectively). However, compared with CR alone, TRE plus CR exhibited no significant benefit on the blood pressure, glucose profile, and lipid profile. Subgroup analysis suggested that early TRE is more effective in weight loss (WMD: −1.42, 95% CI: −1.84 to −1.01, and I^2^: 0%) and improving fat mass (WMD: −1.06, 95% CI: −1.91 to −0.22, and I^2^: 0%) than delayed or broader TRE when combined with CR. Although the combination of TRE and CR can effectively decrease body weight, fat mass, and waist circumference, the long-term effects, particularly those on cardiometabolic risk in participants with chronic cardiovascular disease and diabetes, remain to be explored.

## Introduction

Obesity prevalence has climbed over the past few decades in most nations and has doubled in 73 countries [1]. As a result, obesity is currently regarded as a pandemic, given its gradually rising prevalence and status as an emerging major global public health concern [2]. Growing pieces of evidence reveal that approximately 600 million adults are considered clinically obese, and by 2030, over one billion individuals are expected to suffer from obesity [3]. Effective weight management measures are urgently needed in light of the evidence from epidemiologic studies that obesity is directly linked to an increased risk of chronic diseases, including cardiovascular, neurodegenerative, different types of cancer, and metabolic disorders [4].

Weight loss by lifestyle intervention has been established as fundamental to weight management [5]. Notably, intermittent fasting (IF) has progressively gained popularity as a modified fasting method owing to its clinically significant weight loss effect and ease of application in comparison with daily calorie restriction (CR) [6]. IF became a popular option for numerous populations with excess weight as it neither requires daily calorie tracking nor a limit in the consumption of specific food groups. In contrast, it allows flexible eating patterns at particular times of the day [7]. Furthermore, several clinical studies have proven that IF is superior to continuous dietary restriction in terms of short-term weight loss and body fat improvement [8–11].

Time-restricted eating (TRE), a subtype of IF regimen that calls for a set window of time for eating and fasting within each 24-hour period, has grown in prominence as a creative and workable method of treatment for obesity and metabolic diseases [12]. TRE eliminates the necessity for tracking the caloric intake or count during the eating window, promoting it to be simpler and more convenient [7]. In addition, the circadian rhythm hypothesis progressively enjoys widespread support. The idea highlights that synchronization of feeding and fasting cycles with light and dark circadian rhythms can adjust the efficiency of metabolism and weight reduction [13] as energy homeostasis is controlled by the interaction of peripheral signals with the central nervous system, and any disruption of the circadian rhythms affects weight management and other metabolic processes [14].

Traditionally, TRE was commonly used for dietary intervention in mice laboratory experiments, and earlier animal studies have repeatedly identified that diet plays a vital role in weight loss and improving metabolic parameters [15]. For instance, TRE lowers body weight, improves insulin sensitivity and lipid profile in mice, and alleviates diabetes-induced cognitive impairment by gut microbiota [16]. Massive clinical studies concerning TRE also displayed improvements in body weight, blood pressure, lipid profiles, and insulin resistance in participants [17–20]. Thus, TRE is a practical and well-tolerated dietary strategy over the long term.

Previous meta-analysis publications have investigated the advantages of TRE in various contexts using healthy individuals or participants who are overweight or obese [21–24]. TRE is a potential approach for individuals with excess weight because it considerably decreases body weight and enhances the metabolic parameters related to cardiometabolic health [24]. An updated meta-analysis ascertained that TRE can improve metabolic states in overweight individuals, and a consistent result was found in the metabolic states of normal-weight individuals with a scheduled 16:8 TRE [23]. Allaf et al. illustrated the effect of TRE on weight loss through a detailed systematic review, but the impact of fast diets on clinical outcomes, such as death, myocardial infarction, and heart failure, remains to be elucidated in more detail [21]. Over the past few years, many randomized controlled trials (RCTs) have focused on the benefits of TRE in conjunction with CR. However, lacking meta-analysis pooling particular trials to determine the combined benefits of TRE and CR on body composition and cardiometabolic risk factors. The current systematic review and meta-analysis aimed to further clarify the effects of TRE plus CR on weight loss and cardiometabolic risk.

## Methods

### Protocol and registration

A protocol was developed and followed for all steps of the current systematic review and meta-analysis, and it was registered in INPLASY (https://inplasy.com/) under the record number INPLASY2022100082. The meta-analysis was performed in accordance with the outlines of the Preferred Reporting Items for Systematic Reviews and Meta-analysis Statement (Supplementary Material) [25].

### Search strategy

PubMed, Embase and Cochrane Library were searched from inception to October 18, 2022, for potentially relevant studies without restriction applied to language, publication year, or region using the following search terms: intervention (time-restricted eating, feeding, fasting, or diet) and outcome (blood pressure or diastolic pressure or systolic pressure or diastolic blood pressure or systolic blood pressure or glucose or insulin or homeostatic model assessment for insulin resistance or glucose or insulin or HOMA-IR or HOMA-β or cholesterol or triglyceride or triglycerides or Triacylglycerol or Triacylglycerols or plasma lipid or weight loss or weight losses or weight reduction or weight reductions). The search was updated on January 13, 2023. The complete search terms are described in detail in Supplementary Material. We manually scanned the reference lists of included papers to find gray literature. Furthermore, ClinicalTrials.gov and two gray literature databases were searched for relevant research (OpenGrey.eu and Greylit).

### Eligibility criteria

The inclusion criteria were as follows: (1) study participants over 18 years of age; (2) participants assigned to TRE (for at least 12 h daily) associated with moderate (1200–1500 kcal/d deficit for women and 1500–1800 kcal/d for men) CR; (3) studies reporting outcomes including at least one of the body composition or cardiometabolic risk factors, such as weight loss, fat mass, waist circumference (WC), systolic blood pressure (SBP), diastolic blood pressure (DBP), glucose, insulin, HOMA-IR, HOMA-β, total cholesterol (TC), triglycerides (TG), low-density lipoprotein (LDL); (4) studies with RCTs. Moreover, studies that did not specifically mention TRE and CR but described treatments containing TRE and CR components were considered. Studies enrolled participants taking part in additional weight loss therapies were excluded. This meta-analysis excluded studies without sufficient data and individuals with acute or chronic illnesses that would affect the TRE treatment. If studies comprised the same trials, we analyzed the study with the most extended duration or comprehensive information.

### Data extraction

Study selection was performed in two phases: an initial title and abstract screening followed by a complete text examination of papers for suitability in this research. Two review authors (JCS and ZTT) independently assessed the eligibility of the papers, and any discrepancies were settled by a third investigator (GQ). The full texts of potentially eligible articles were obtained for further evaluation. Data extraction was performed systematically by two authors (CJH and CLZ) using data extraction sheets, and discrepancies were addressed by consensus. The extracted data included the first author, publication year, country, duration of follow-up, study design, sample size, demographic characteristics of the participants, mean weight, mean body mass index (BMI), body composition, fasting glucose, insulin, blood pressure, HOMA-IR, HOMA-β, and lipid profile. We reached out to the authors of articles that lacked relevant data.

All outcomes were continuous variables, and thus, the mean changes and corresponding standard deviation (SD) values were extracted in both intervention and control groups. The changes in mean and SD were calculated using the correlation coefficient approach suggested in the Cochrane Handbook [26] if studies supplied the mean and SD values at baseline and post-intervention. Studies without SD were converted using documented methods employing standard error of measurement or, if available, confidence interval (CI). WebPlotDigitizer (version 4.6) was used to extract the TC, TG, and LDL data from graphical representations in the study by Thomas et al. [27].

### Risk of bias and certainty of evidence assessment

Two reviewers (JCS and ZTT) independently evaluated the risk of bias in selected studies using the revised Cochrane risk of bias tool for randomized trials (ROB2) [28]. The biases of the included studies constituted six domains, such as selection, performance, detection, attrition, reporting, and overall biases. For these domains, we assessed the risk of bias in randomized, parallel-group trials by examining the randomization process, deviations from intended interventions, mising outcome data, measurement of the outcome, and selection of the reported result. When evaluating the risk of bias for crossover trials, we introduced an additional aspect—bias arising from period and carryover effects. The risk associated with each domain was classified as low, some concerns, or high. Discrepancies were resolved by a senior investigator (GQ).

The Grading of Recommendations Assessment, Development, and Evaluation (GRADE) framework [29] was used to categorize the evidence quality of each outcome into very low, low, moderate, and high levels by two writers (CLZ and CJH) independently. Disagreements were settled by a third reviewer (HLH). The RCT studies were initially rated as high quality and then downgraded or upgraded depending on predetermined criteria, including the risk of bias, consistency of results across studies, directness, the precision of results, likelihood of publication bias, large effect, dose-response gradient, or all other plausible confounders that attenuate the pooled risk estimates.

### Data analysis

Stata Statistical Software version 16.0 (StataCorp, College Station, TX, USA) was used for the analysis. Two-sided P values were evaluated for significance at an alpha level of 0.05. Differences between the final and baseline mean values of each outcome were reported as change values and analyzed. The effect size was determined by the weighted mean difference (WMD) between these differences. The I^2^ statistic was used to quantify the degree of heterogeneity between studies (0–25% for low heterogeneity, 25–50% for moderate heterogeneity, 50–75% for substantial heterogeneity, and 75%–100% for high heterogeneity). Considering the likely high heterogeneity because of diverse clinical and methodological factors in included studies, the random-effects model of Dersimonian and Laird was used to calculate the pooled WMD with 95% CI for the TRE combined with CR and their effect on weight loss and cardiometabolic risk. Sensitivity analyses by sequential deletion of trials were used to investigate results with moderate or high levels of heterogeneity to determine the heterogeneity source.

Considering the different subtypes of TRE, subgroup analyses were performed based on broader TRE (unclear due to self-selective eating windows for ad libitum) with CR, early TRE (eTRE) with CR, and delayed TRE (dTRE) with CR. Subgroup analyses were also conducted to determine the subgroup difference after stratifying for daily fasting to eating ratios (16:8 vs. 14:10 vs. 12:12), mean baseline BMI (overweight vs. obesity), duration (short-term vs. long-term), and study location (Europe vs. South America vs. North America vs. Asia). Short-term study duration was defined as less than 12 weeks, whereas a period of 12 weeks or longer was deemed a long-term study duration. To evaluate potential publication bias, we assessed funnel plot asymmetry by visualization and performed a trim-and-fill analysis if publication bias was detected. Additionally, we used Egger’s tests to identify small-study effects.

## Results

### Search results

**Fig. 1:**
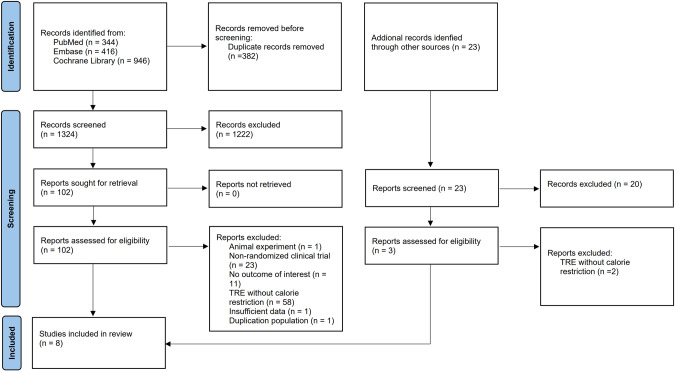
Flow diagram. Flow diagram showing search strategy and inclusion and exclusion of studies for meta-analysis.

After removing duplicates, 1347 records were retrieved during the initial and updated search. The full texts of 105 papers were obtained for further assessment based on titles and abstract screening. Eight publications covered 579 participants and were qualified for data extraction and quantitative analysis [27, 30–36]. A study by Amodi et al. [37] was excluded due to the absence of pertinent data and contact information for correspondence, as well as the remaining 96 were eliminated because they failed to meet the predetermined inclusion criteria. Two publications from the same study were acquired, and we analyzed articles with a long follow-up period to determine the long-term effects of TRE with CR [30, 38]. Figure 1 depicts the flow of studies through the review.

### Characteristics of the included studies

All eight articles included individuals who were overweight or obese, 147 of whom were male. Only one study focused on participants with diabetes [30]. Studies from China [33, 35], Brazil [31, 36], and the United States [27, 32, 34], in addition to one study from the Czech Republic [30], were considered. Except for one paper that employed a randomized crossover design [30], all the trials considered were RCTs. Queiroz et al. [36] employed three interventions: a group with CR, a group with eTRE plus CR, and the last group with dTRE plus CR. The crossover study [30] compared the effect of six versus two meals a day, and thus, the study was classified into eTRE with 14 h fasting cycle. Two trials [31, 33] restricted participants to women, four studies [27, 32, 34, 36] virtually exclusively recruited women, and two others [30, 35] recruited a gender-balanced group. Table 1 summarizes the detailed participant and study characteristics.

**Table 1:** Characteristics of included studies.

Study	Study design	Participants	Study Duration	Intervention	Control group	Simple size	Age (year)	Sex (F/M)	Body composition	Blood pressure (mmHg)	Fasting glucose (mg/dl)	Lipid level (mg/dl)
Kahleova, 2014	Randomized crossover	Patients with diabetes	12w	eTRE (14:10) + CR (500 kcal/d deficit)	CR (500 kcal/d deficit)	54	59.4 ± 7.0	25/29	Weight: 94.1 ± 15.5(kg)	SBP: 140.0 ± 14.0 DBP: 85.0 ± 8.0	UN	UN
Pureza, 2020	RCT	Women with obesity	12 m	Broader TRE (12:12) + CR (500-1000 kcal/d deficit)	CR (500-1000 kcal/d deficit)	58	31.0 ± 7.0	58/0	Weight: 80.8 ± 11.7 (kg) WC: 101.0 ± 10.3(cm)	SBP: 125.7 ± 13.5 DBP: 86.3 ± 11.8	UN	UN
Peeke, 2021	RCT	Patients with obesity	8w	eTRE (14:10) + CR (500–1000 kcal/d deficit)	TRE (12:12) + CR (500–1000 kcal/d deficit)	78	44.0 ± 11.0	68/10	Weight: 123.4 ± 20.6 (kg)	UN	103.2 ± 30.8	UN
Lin, 2021	RCT	Middle-aged women with overweight or obesity	8w	Broader TRE (16:8) + CR (1400 kcal/d)	CR (1400 kcal/d)	63	54.2 ± 7.9	63/0	Weight: 65.9 ± 9.2 (kg) WC: 88.4 ± 8.9 (cm)	SBP: 121.2 ± 14.4 DBP: 73.2 ± 10.8	88.9 ± 8.1	TC: 187.5 ± 34.7 TG: 106.1 ± 43.2 LDL: 108.0 ± 529.2
Jamshed, 2022	RCT	Patients with obesity	14w	eTRE (16:8) + CR (500 kcal/d deficit)	CR (500 kcal/d deficit)	90	43.0 ± 11.0	78/18	Weight: 108.8 ± 20.6(kg) Fat mass: 52.5 ± 14.4(kg)	SBP: 124.0 ± 13.0 DBP: 81.0 ± 9.0	105.0 ± 16.0	TC: 203.0 ± 38.0 TG: 117.0 ± 64.0 LDL: 118.0 ± 28.0
Queiroz, 2022	RCT	Patients with overweight or obesity	8w	eTRE (16:8) + CR (25 % energy deficit) dTRE (16:8) + CR (25 % energy deficit)	CR (25 % energy deficit)	48	30.0 ± 6.0	31/6	Weight: 82.9 ± 12.8(kg)	SBP: 111.0 ± 12.0 DBP: 77.0 ± 11.0	98.0 ± 8.0	TG: 95.0 ± 33.0
Liu, 2022	RCT	Patients with obesity	12 m	eTRE (16:8) + CR (man: 1500-1800kcal/day, woman: 1200- 1500 kcal/day)	CR (man: 1500-1800kcal/day, woman: 1200- 1500 kcal/day)	139	31.9 ± 9.1	71/68	Weight: 88.2 ± 11.6(kg) Fat mass: 33.1 ± 6.8(kg) Lean mass: 51.0 ± 8.5	SBP: 125.0 ± 12.1 DBP: 73.8 ± 9.5	91.5 ± 14.4	TC: 196.3 ± 35.4 TG: 133.8 ± 61.0 LDL: 130.1 ± 31.8
Thomas, 2022	RCT	Patients with BMI of 27 to 45 kg/m^2^	12w	eTRE(14:10) + CR(10-35% energy deficit)	CR (10-35% energy deficit)	85	38 ± 7.8	69/16	Weight: 94.8 ± 18.2 Fat mass: 40.7 ± 11.5	UN	UN	UN

*BMI* Body mass index, *RCT* Randomized controlled trial, *TRE* Time-restricted eating, *eTRE* early time-restricted eating, *dTRE* Delayed time-restricted eating, *CR* calorie restriction, *F* Female, *M* Male, *WC* Waist circumference, *SBP* Systolic blood pressure, *DBP* Diastolic blood pressure, *TC* Total cholesterol, *TG* Triglycerides, *LDL* low-density lipoprotein.

### Risk of bias assessment

**Fig. 2:**
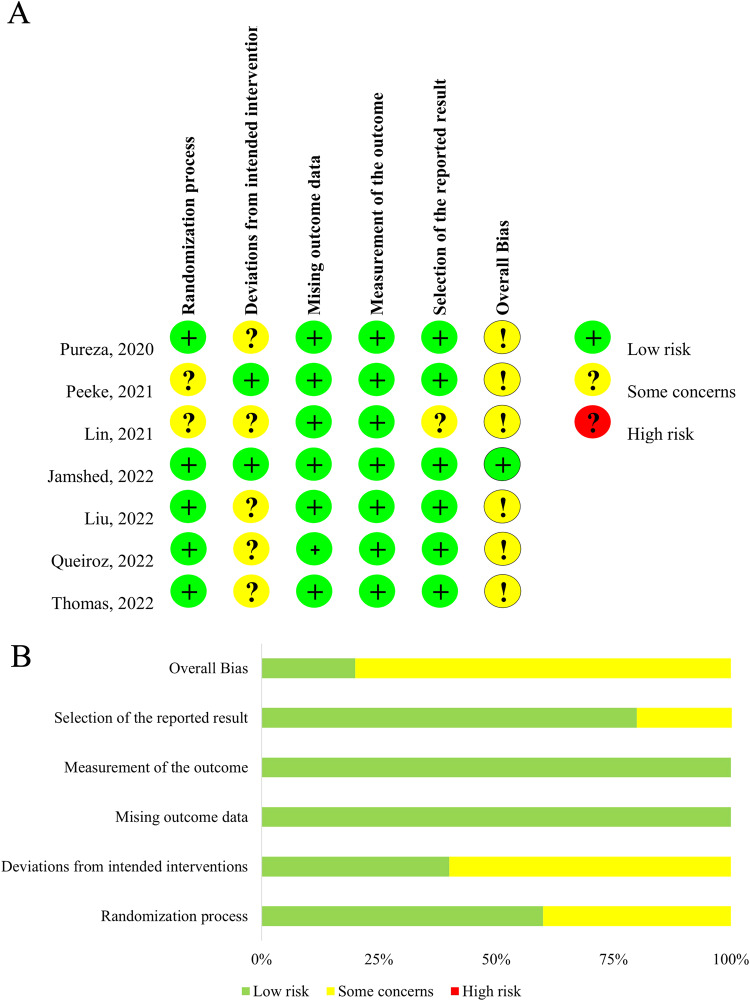
Risk of bias assessment in the randomized parallel-arm studies. **A** Summary of risk of bias. **B** Quality assessment percentages in the meta-analysis.

**Fig. 3:**
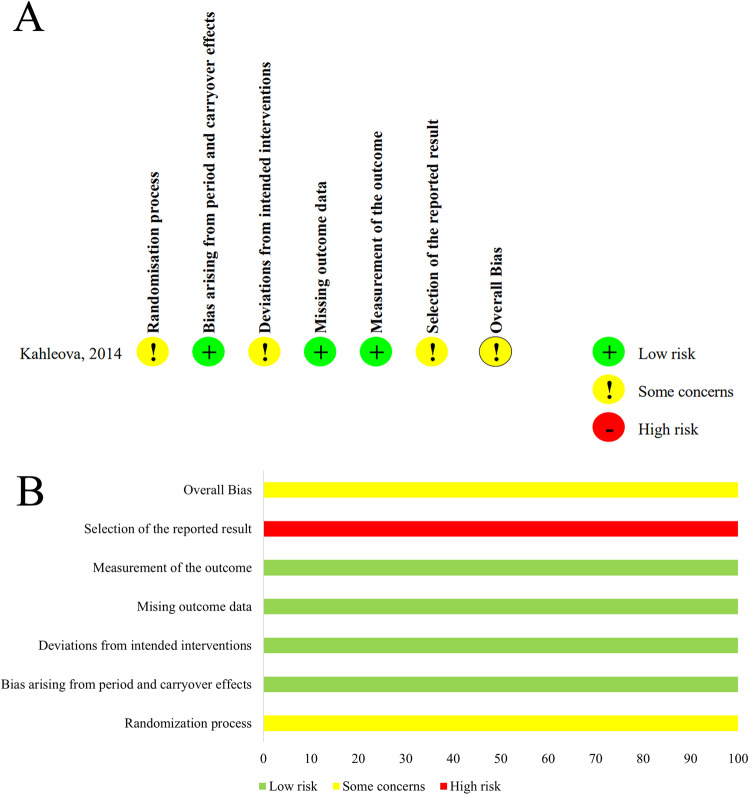
Risk of bias assessment in the randomized cross-over study. **A** Summary of risk of bias. **B** Quality assessment percentages in the meta-analysis.

A version of ROB2 for individually randomized, parallel-group trials was applied to evaluate seven parallel-arm designed studies, and the results are displayed in Fig. 2. Only one study [34] had a low risk of bias, and the other seven [27, 31–33, 35, 36] had some concerns. The risk of bias was some concerns in two articles [32, 33] for lack of information about the randomization process, in five studies [27, 31, 33, 35, 36] for deviations from intended interventions, and in one publication [36] for missing outcome data. One crossover design research [30] was assessed by a version of ROB2 for crossover trials. The trial had some concerns about bias, given the absence of a randomization process, deviations from intended interventions and selection of the reported result (Fig. 3).

### Efficacy of TRE with CR on weight loss and body composition

**Fig. 4:**
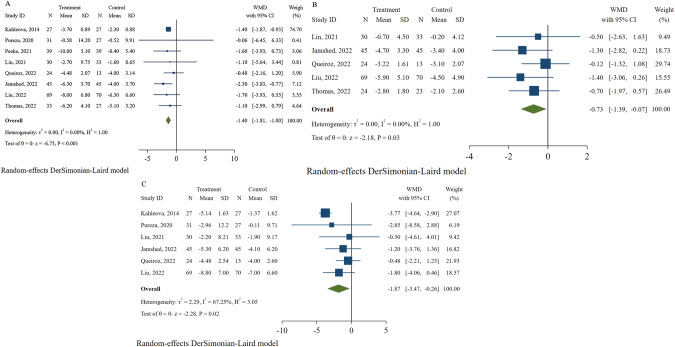
Effects of time-restricted eating with calorie restriction on body weight and body composition. **A** Body weight. **B** Waist circumference. **C** Fat mass.

Eight studies [27, 30–36] with 579 participants reported weight loss as an outcome. Compared with the control group, participants assigned to the TRE combined with the CR group showed a significant decrease in weight (WMD: −1.40, 95% CI: −1.81 to −1.00, and I^2^: 0%; Fig. 4A). Despite the removal of a study [30] with the highest weight (73.51%), a similar result was obtained (WMD: −1.42, 95% CI: −2.23 to −0.61, and I^2^: 0%; Fig. S1). Although the asymmetric funnel plot (Fig. S2) indicates the existence of a possible publication bias, trim-and-fill analysis yielded a WMD of −1.47 with 95%CI ranging from −1.86 to −1.07 (Fig. S3). Additionally, Egger’s test detected no small-study effects (*P* = 0.841).

The changes in fat mass were recorded in five studies (376 individuals) [27, 33–36]. The TRE combined with CR decreased fat mass without significant heterogeneity among studies (WMD: −0.73, 95% CI: −1.39 to −0.07, and I^2^: 0%; Fig. 4B). Six studies [30, 31, 33–36] involving 441 participants showed changes in WC from baseline to endpoint. Compared with the control group, the TRE with CR group showed a significantly reduced WC (WMD: −1.87, 95% CI: −3.47 to −0.26, and I^2^: 67.25%; Fig. 4C), but substantial heterogeneity was observed among studies. The heterogeneity decreased when the study by Kahleova et al. [30] was removed, and the result changed (WMD: −1.04, 95% CI: −2.18 to 0.10, and I^2^: 0%).

### Efficacy of TRE with CR on cardiometabolic risk

**Fig. 5:**
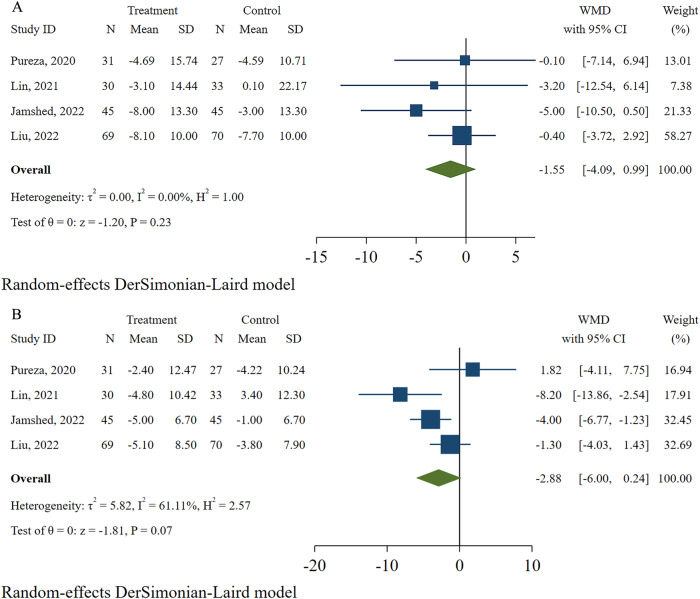
Effects of time-restricted eating with calorie restriction on blood pressure. **A** Systolic blood pressure. **B** Diastolic blood pressure.

Four studies [31, 33–36] with 350 individuals focused on the effect on blood pressure. Compared with the control group, the TRE combined with the CR group revealed a lowered SBP without showing significant difference and heterogeneity (WMD: −1.55, 95% CI: −4.09 to 0.99, and I^2^: 0%; Fig. 5A). The result for DBP was similar but with a significant heterogeneity among studies (WMD: −2.88, 95% CI: −6.00 to 0.24, and I^2^: 61.11%; Fig. 5B). Sensitivity analysis unveiled a degraded heterogeneity without changes in statistical significance after the removal of the study by Lin et al. [33] (WMD: −1.90, 95% CI: −4.65 to 0.85, and I^2^: 47.49%).

**Fig. 6:**
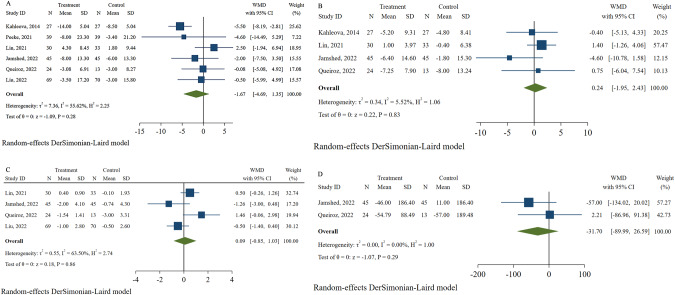
Effects of time-restricted eating with calorie restriction on glucose profile. **A** Fasting glucose. **B** Insulin. **C** HOMA-IR. **D** HOMA-β.

Six studies [30, 32–36] with 461 participants recorded changes in fasting glucose concentration with TRE plus CR. There was no significant difference in fasting glucose between groups with a moderate heterogeneity (WMD: −1.67, 95% CI: −4.69 to 1.35, and I^2^: 55.62%; Fig. 6A), and the result was consistent without heterogeneity when the study by Kahleova et al. [30] was removed (WMD: −0.04, 95% CI: −2.48 to 2.41, I^2^: 0%). For insulin, the pooled analysis of four studies [30, 33, 34, 36] with 244 individuals suggested no differences between the intervention and control groups (WMD: 0.24, 95% CI: −1.95 to 2.43, and I^2^: 5.52%; Fig. 6B). Moreover, HOMA-IR and HOMA-β were investigated in four studies [33–36] (329 participants) and two articles [34, 36] (127 participants) respectively, but no statistically significant differences were found in either of the two outcomes (WMD: 0.09, 95% CI: −0.85 to 1.03, and I^2^: 63.50%; WMD: −31.70, 95% CI: −89.99 to 26.59, and I^2^: 0%, respectively; Fig. 6C, D). A nonsignificant difference was observed in the HOMA-IR after the sequential deletion of trials without a marked change in heterogeneity.

**Fig. 7:**
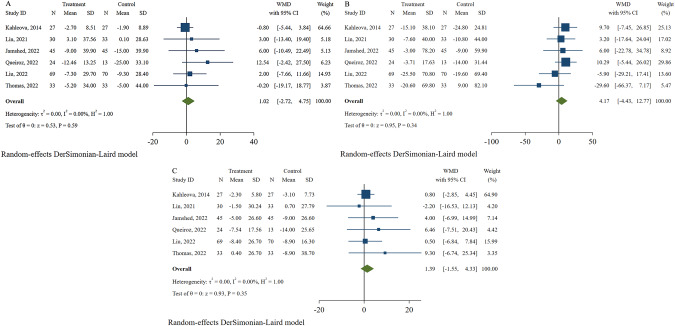
Effects of time-restricted eating with calorie restriction on lipid profile. **A** Total cholesterol. **B** Triglyceride. **C** Low-density lipoprotein.

Changes in lipid profiles were reported by six studies [27, 30, 33–36], with 449 participants reported. The results showed that TRE plus CR had no beneficial effects of TRE plus CR on TC (WMD: 1.02, 95% CI: −2.72 to 4.75, and I^2^: 0%; Fig. 7A), TG (WMD: 4.17, 95% CI: −4.43 to 12.77, and I^2^: 0%; Fig. 7B), or LDL (WMD: 1.39, 95% CI: −1.55 to 4.33, and I^2^: 0%; Fig. 7C) without significant heterogeneity.

### Subgroup analyses

Subgroup analysis using intervention subtypes was conducted, and eTRE plus CR significantly reduced body weight and fat mass compared with CR alone with WMD of −1.42 kg (95% CI: −1.84 to −1.01; I^2^: 0%) and −0.74 kg (95% CI: −1.47 to −0.02 and I^2^: 0%; Table 2), respectively. However, no significant effects were found on weight loss and fat mass loss in the broader TRE with CR and dTRE with CR groups. Moreover, WC reduced nonsignificantly in all three groups.

**Table 2:** Subgroup analysis of weight loss and body composition.

Outcomes of interest	Weight loss			WC			Fat mass		
	Studies, no.	WMD (95% CI)	Heterogeneity I2, %	Studies, no.	WMD (95% CI)	Heterogeneity I2, %	Studies, no.	WMD (95% CI)	Heterogeneity I2, %
Intervention									
eTRE+CR	6	−1.42 (−1.84, −1.01)	0	4	−1.88 (−3.83, 0.08)	79.39	4	−0.74 (−1.47, −0.02)	0
dTRE+CR	1	−0.80 (−2.86, 1.26)	-	1	−0.80 (−3.02, 1.42)	-	1	−0.50 (−1.99, 0.99)	-
TRE + CR	2	−0.75 (−4.45, 2.95)	0	2	−1.22 (−4.67, 2.23)	0		−0.50 (−2.63, 1.63)	-
Daily fasting to eating ratio									
16:8	4	−1.50 (−2.49, −0.52)	0	4	−0.97 (−2.13, 0.20)	0	4	−0.74 (−1.51, 0.02)	0
14:10	3	−1.39 (−1.84, −0.94)	0	1	−3.77 (−4.64, −2.90)	-	1	−0.70 (−1.97, 0.57)	-
12:12	1	−0.06 (−6.45, 6.33)	-	1	−2.85 (−8.58, 2.88)	-	-	-	-
Mean baseline BMI									
BMI > 30 kg/m2	7	−1.41 (−1.82, −1.00)	0	5	−2.03 (−3.74, −0.31)	71.37	4	−0.75 (−1.44, −0.06)	0
BMI ≥ 25 kg/m2 and<30 kg/m2	1	−1.10 (−5.64, 3.44)	-	1	−0.30 (−4.61, 4.01)	-	1	−0.50 (−2.63, 1.63)	-
Study location									
Asia	2	−1.58 (−3.58, 0.42)	0	2	−1.48 (−3.48, 0.53)	0	2	−1.06 (−2.37, 0.25)	0
Europe	1	−1.40 (−1.87, −0.93)	-	1	−3.77 (−4.64, −2.90)	-	-	-	-
North America	3	−1.78 (−2.84, −0.72)	0	1	−1.20 (−3.76, 1.36)	-	2	−0.95 (−1.92, 0.03)	0
South America	2	−0.45 (−2.08, 1.17)	0	2	−0.68 (−2.33, 0.98)	0	1	−0.12 (−1.32, 1.08)	-
Duration									
<12w	3	−0.88 (−2.19, 0.42)	0	2	−0.46 (−2.06, 1.15)	0	2	−0.21 (−1.26, 0.83)	0
>12w	5	−1.46 (−1.89, −1.03)	0	4	−2.69 (−4.17, −1.20)	44.69	3	−1.06 (−1.91, −0.22)	0

*BMI* Body mass index, *TRE* Time-restricted eating, *eTRE* early time-restricted eating, *dTRE* Delayed time-restricted eating, *CR* calorie restriction, *WC* Waist circumference, *WMD* Weight mean difference.

In the subgroups with various daily fasting-to-eating ratios (16:8, 14:10, and 12:12), the weight was reduced significantly in the 16:8 (WMD: −1.50, 95% CI: −2.49 to −0.52, and I^2^: 0%; Table 2) and 14:10 groups (WMD: −1.39, 95% CI: −1.84 to −0.94, and I^2^: 0%; Table 2), but a nonsignificant decrease was recorded in the 12:12 group (WMD: −0.06, 95% CI: −6.45 to 6.33; Table 2). No fat mass outcome was included in the 12:12 group, but fat mass decreased without a significant difference in the other two groups (WMD: −0.74, 95% CI: −1.51 to 0.02, and I^2^: 0%; WMD: −0.70, 95% CI: −1.97 to 0.57; Table 2). As for WC, the 14:10 TRE combined with the CR group showed a significantly improved WC (WMD: −3.77, 95% CI: −4.64 to −2.90; Table 2), but the results were nonsignificant in the 12:12 and 16:8 groups.

TRE plus CR effectively reduced body weight (WMD: −1.41, 95% CI: −1.82 to −1.00, and I^2^: 0%; Table 2), fat mass (WMD: −0.75, 95% CI: −1.44 to −0.06, and I^2^: 0%; Table 2) and WC (WMD: −2.03, 95% CI: −3.74 to −0.31, and I^2^: 71.37%; Table 2) compared with the control group in obesity subgroup (mean baseline BMI > 30 kg/m^2^). Conversely, in overweight (mean baseline BMI ≥ 25 and <30 kg/m^2^) participants, weight loss was nonsignificant, and similar outcomes were observed in the fat mass and WC.

Subgroup analysis based on regions (Asia, Europe, North America, and South America) suggested that studies conducted in Europe and North America found beneficial effects of TRE with CR on weight loss (WMD: −1.40, 95% CI: −1.87 to −0.93; WMD: −1.78, 95% CI: −2.84 to−0.72, and I^2^: 0%, respectively; Table 2). Otherwise, no significant difference was found in terms of fat mass. The study by Kahleova et al. [30] in Europe showed a significant decrease in WC (WMD: −3.77, 95% CI: −4.64 to −2.90, and I^2^: 0%; Table 2), but studies conducted in other regions did not detect differences.

Subgroup analysis was conducted based on different durations to investigate the long-term effects of combined TRE and CR. The results showed that the body weight was reduced in participants who received a long-term period (>12 weeks) of intervention (WMD: −1.46, 95% CI: −1.89 to −1.03, and I^2^: 0%; Table 2), but a nonsignificant decrease was observed in those who were scheduled for short-term intervention duration (<12 weeks). Consistent fat mass and WC outcomes were observed with the body weight (Table 2).

### GRADE assessment

Table S1 presents the GRADE assessment results. Among the 12 outcomes analyzed, weight loss, SBP, glucose, TC, TG, and LDL were classified as low quality, and the other six outcomes (fat mass, WC, DBP, insulin, HOMA-IR, HOMA-β) were graded as very low.

## Discussion

This systematic review and meta-analysis of 8 trials involving 579 participants revealed that participants who follow a combined TRE and CR regimen efficiently lose body weight and substantially reduce their WC and fat mass. However, no changes were observed in the other outcomes, such as SBP, DBP, fasting glucose, insulin, HOMA-IR, HOMA-β, and lipid profile values (TC, TG, and LDL). The funnel plot and Egger’s test revealed neither publication bias nor small-study effects on body weight in the selected papers. One study presented a low risk of bias, whereas seven studies raised some concerns. The GRADE evaluation rated six of the twelve outcomes from the current study as low quality, and the remaining six were classified as very low quality.

The present study result matched the earlier trial’s conclusions that proposed TRE with CR as a successful measurement tool to improve body composition [39]. Several lines of evidence consider the potential role of rhythmic creatine-mediated thermogenesis in the metabolic advantages of time-restricted meals [40]. Furthermore, mounting proof validates that TRE promotes a fuel switch from glucose to fatty acids [41], which is associated with enhanced expression of oxidative metabolic genes in adipose tissue, improved energy consumption, and prevention of metabolic diseases without modification of food intake. These mechanisms can be responsible for the findings observed in the present study; that is, participants who experienced TRE with CR exhibited excellent body composition improvement benefits compared with individuals who underwent CR alone. Notably, in the present meta-analysis, the heterogeneity of WC disappeared when the study by Kahleova et al. [30] was excluded, and the results showed a nonsignificant difference, indicating the robustness of the findings on WC was mainly influenced by the excluded research.

Subgroup analysis revealed increased body weight loss and fat mass reduction tendencies in participants with eTRE plus CR intervention compared with CR alone, but no body weight or fat mass benefits was found in the subgroups of dTRE plus CR, broader TRE plus CR. The analysis results should be interpreted cautiously because dTRE was only exploited in one study [36]. The subgroup analysis results for WC based on intervention measures revealed no significant difference between groups, indicating that body weight but not WC is the most sensitive parameter. Contrary to previous meta-analyses [23], participants with obesity in the present study benefited from the combination of TRE and CR, and they showed significant body weight reduction and body composition improvement. Such a discrepancy may be explained in part by the scarce studies in the meta-analysis by Liu et al. [23], in which only one study [42] involving 49 participants confirmed the positive effects of TRE on obesity; in addition, the combination of opposing data may offset the benefits of TRE. A review on TRE resulting in reduced body weight in individuals with obesity by imposing eating/fasting cycles hypothesized that it would restore robust circadian rhythms, which may support the novel findings in our work [43]. Subgroup analysis of duration showed that long-term intervention by TRE plus CR is more effective in weight loss and body composition improvement than its short-term counterpart. Although the long-term group showed a significant effect, two of the four included studies [31, 35] with a 12-month follow-up period exhibited no changes in body weight, fat mass, or WC. Future long-term trials with large subject populations will be necessary to further determine the long-term benefits of TRE plus CR on body composition [44].

Subgroup analysis based on diverse daily fasting-to-eating ratios demonstrated that TRE combined with CR had significant positive effects on body weight if the fasting window was 14 h or more, whereas no significant difference was discovered in fat mass and WC among any subgroups, except for the 14:10 group in WC [30]. However, the WC outcome of the 14:10 group may need to be more reliable, given the small number of included studies. Moreover, the analysis results of studies supported that TRE combined with CR is more effective in reducing body weight when conducted in developed countries (Europe and North America) as opposed to developing countries (Asia or North America), consistent with the epidemiologic research of obesity prevalence in developed countries [45]. This phenomenon may be associated with the high popularity of health education about obesity and its complications in these areas. The subgroup analysis results based on regions showed no changes in WC and fat mass, except for the Europe subgroup of one study [30], where a significant decrease in WC was observed.

The TRE regimen did not significantly benefit blood pressure compared with the CR regimens. The sensitivity analysis of DBP detected a moderate heterogeneity when the study by Lin et al. [33] was removed but showed no change in the outcome, proving the robustness of the result. A large, 12-week RCT compared TRE (with an 8 h eating window from 12:00 PM to 8:00 PM) with consistent meal timing (eating three structured meals per day) and offered the same conclusion as the present research [46]. Nevertheless, a 5-week randomized crossover trial with positive results proposed that in comparison with the controlled schedule, eTRE considerably reduced the morning levels of SBP and DBP [47]. The controversy is possibly attributed to the difference in insulin and a statement that the reduction in insulin levels can improve blood pressure [48], as well as the fact that we noticed no differences in insulin levels in the study by Lowe et al. [47] or the present meta-analysis. Furthermore, although the analysis revealed a significantly reduced weight, according to the guide, the change in body weight was relatively mild to induce a significant difference in blood pressure [49].

The current meta-analysis showed that TRE did not provide extra benefits on glycemic and lipid profiles compared with daily CR. Sensitivity analysis showed a similar result in glucose levels between groups when the study causing heterogeneity was excluded. As mentioned in the current review, isocaloric TRE, particularly in individuals with prediabetes, improves fasting insulin and insulin resistance independent of weight loss [50], but with a controversy on the glucose profile in participants scheduled for ad libitum TRE who do not have diabetes [51–54]. The result provides evidence against the positive conclusion of a previous meta-analysis involving 19 studies, which showed that TRE could lower fasting blood glucose, HOMA-IR, and lipid spectrum of TG, TC, and LDL-C in overweight individuals [24]. Diverse population characteristics in the included studies may explain the conflicting conclusion in the present research and that of Moon et al. But it is noteworthy that among the latter, neither significant lipid nor glucose profile changes were observed in the subgroup analysis based on healthy subjects, and the sensitivity analysis did not turn up any outline studies, indicating some skepticism about the robustness of the findings. The blood lipid outcomes in the present meta-analysis contradict the hypothesis put forward in a review that TRE may contribute to favorable changes in some aspects of the lipid profile concurrent with a simultaneous reduction in body weight, despite insufficient evidence [55]. A possible explanation for this may be that the studies included were intended to concentrate on the reduction effect of TRE combined with CR on body weight rather than improving blood lipids. Consequently, the slightly different inclusion criteria resulted in an imbalanced baseline serum lipid level of participants in several studies that excluded participants with a history of cardiovascular disease or diabetes [27, 36]. Two trials did not recruit individuals taking medications for blood sugar or blood lipids [30, 33]. More well-designed large sample studies are desiderated to ensure the scientific, objective, and reliable conclusions of the trials in future clinical research to identify the effectiveness of TRE plus CR on glucose and lipid profiles.

We acknowledge that the present study has some limitations. The main limitation of the present meta-analysis was the low or very low quality evidence of study outcomes classified by the GRADE tool, which was primarily attributed to the risk of bias that the nature of behavioral intervention by a fixed ratio of fasting and eating periods, preventing it from blinding the participants. Nevertheless, the study results were all objective measures that were slightly affected by subjective dimension and influenced the outcomes mildly regardless of whether the participants were informed of allocation results. Second, several results displayed varied heterogeneity because of the TRE subtype, various fasting and eating duration ratios, and different baseline body weights. Subgroup analyses were carried out to investigate the combined effects of TRE and CR on specific subgroups. Meanwhile, sensitivity analysis was also conducted to determine the source of heterogeneity in outcomes with substantial and high heterogeneity. Third, the representativeness of participants was impaired by rigorous inclusion criteria, with several trials eliminating individuals under treatment for diabetes and cardiovascular disease. As a result, the findings cannot be used to generalize clinical participants, especially those with chronic diseases receiving treatment. The quality of evidence and dependability of the findings are expected to increase in future long-term studies with representativeness and a large sample of participants.

## Conclusion

Our systematic review and meta-analysis demonstrated that calorie-intake restriction with time restriction could significantly decrease body weight, fat mass, and WC. Although the combination of TRE and CR did not benefit cardiometabolic risk factors (blood pressure, glucose, or lipid profile), the results should be interpreted with caution because of the specificity of the included population. The present study results highlight the need for well-designed, large-sample, and long-term studies to improve the evidence quality for the effect of TRE combined with CR on participants with chronic cardiovascular disease and diabetes.

### Supplementary information

supplementary figures

supplemental tables

Spreadsheet for risk of bias assessment process

search terms

PRISMA checklist

## Data Availability

Because this is a meta-analysis, all of the data included in this study can be found in the included references.
