# Correction: Time-restricted eating with calorie restriction on weight loss and cardiometabolic risk: a systematic review and meta-analysis

**DOI:** 10.1038/s41430-023-01341-4

**Published:** 2023-09-08

**Authors:** Jing-Chao Sun, Zhen-Tao Tan, Chao-Jie He, Hui-Lin Hu, Chang-Lin Zhai, Gang Qian

**Affiliations:** 1 Department of Cardiology, The Affiliated Hospital of Jiaxing University, Jiaxing, Zhejiang 314000 China; 2 Jiaxing University Master Degree Cultivation Base, Zhejiang Chinese Medical University, Jiaxing, Zhejiang 314000 China

**Keywords:** Obesity, Weight management, Nutrition, Lifestyle modification

Correction to: *European Journal of Clinical Nutrition* 10.1038/s41430-023-01311-w, published online 24 July 2023

In the original article, the funding grant number ‘LGF21H020006’ was given incorrectly as ‘LGF21H090017’. The original article has been corrected.

