# Supplementary material for: Time-restricted eating with calorie restriction on weight loss and cardiometabolic risk: a systematic review and meta-analysis
Source: Eur J Clin Nutr. 2023 Jul 24;77(11):1014–25. doi: 10.1038/s41430-023-01311-w (PMC10630127; doi:10.1038/s41430-023-01311-w)
Supplement: Supplementary file 1 — supplementary figures [file 41430_2023_1311_MOESM1_ESM.docx]

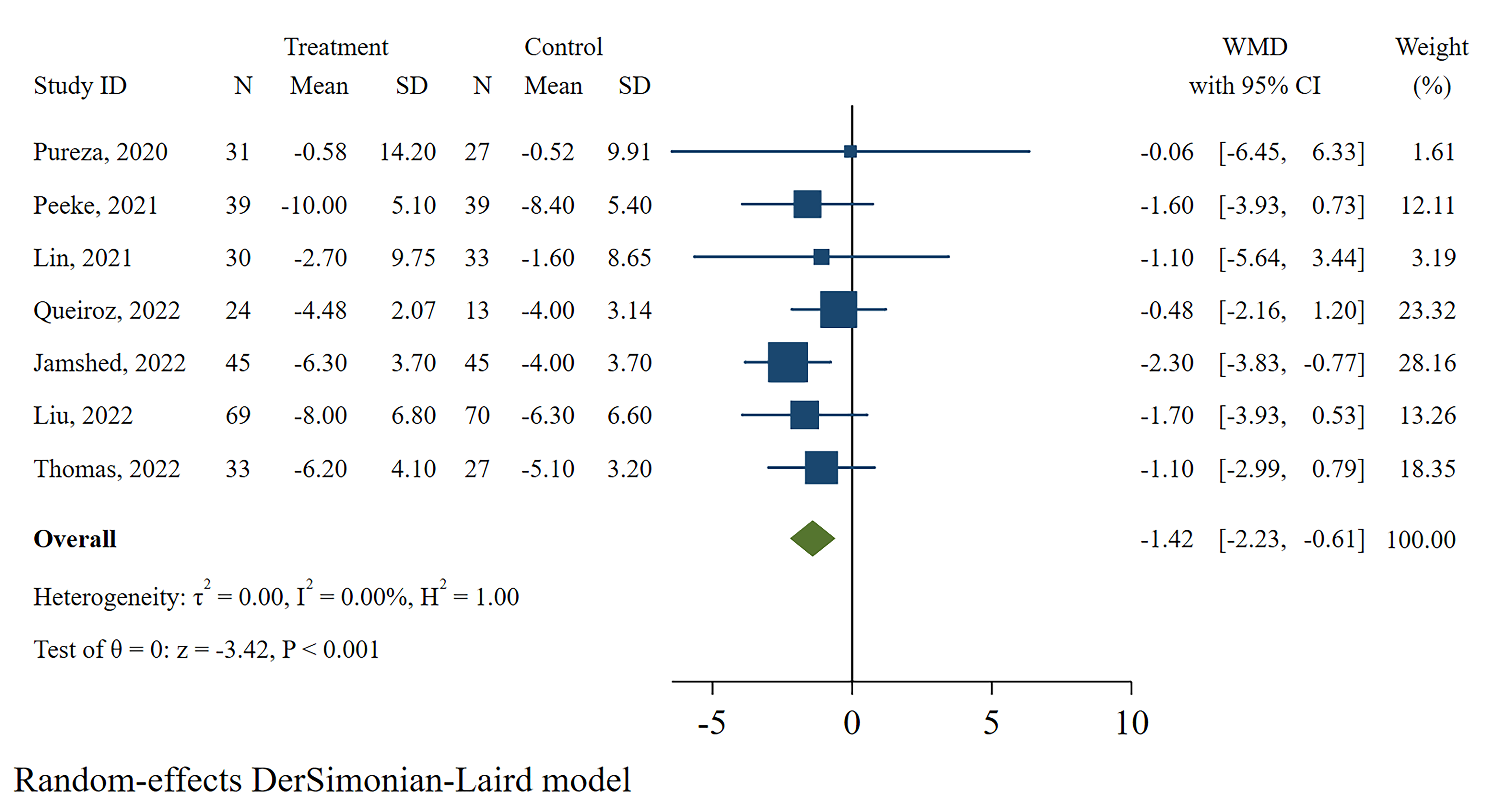


Figure S1. Sensitive analysis in weight loss excluding the study by Kahleova et al.


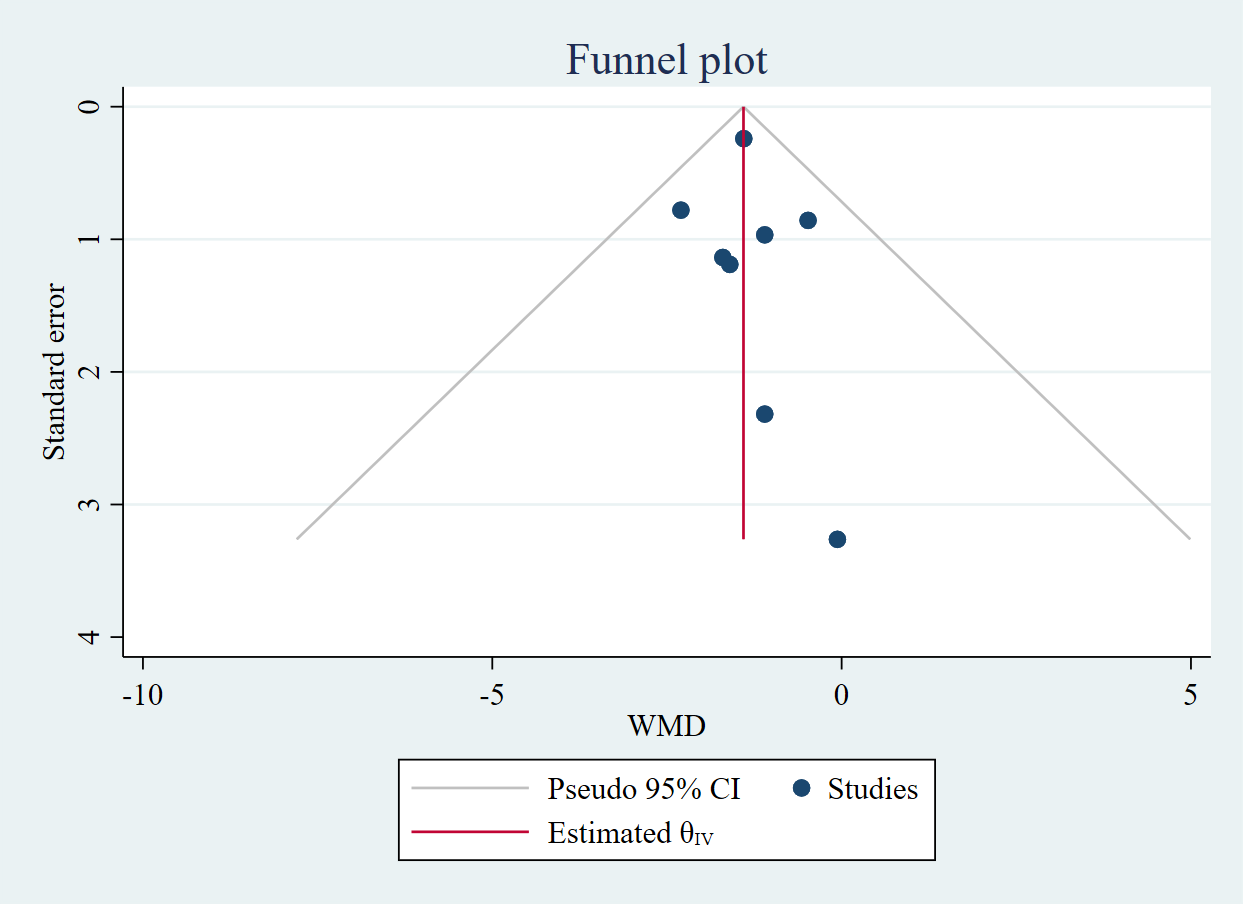


Figure S2. Funnel plots for weight loss.


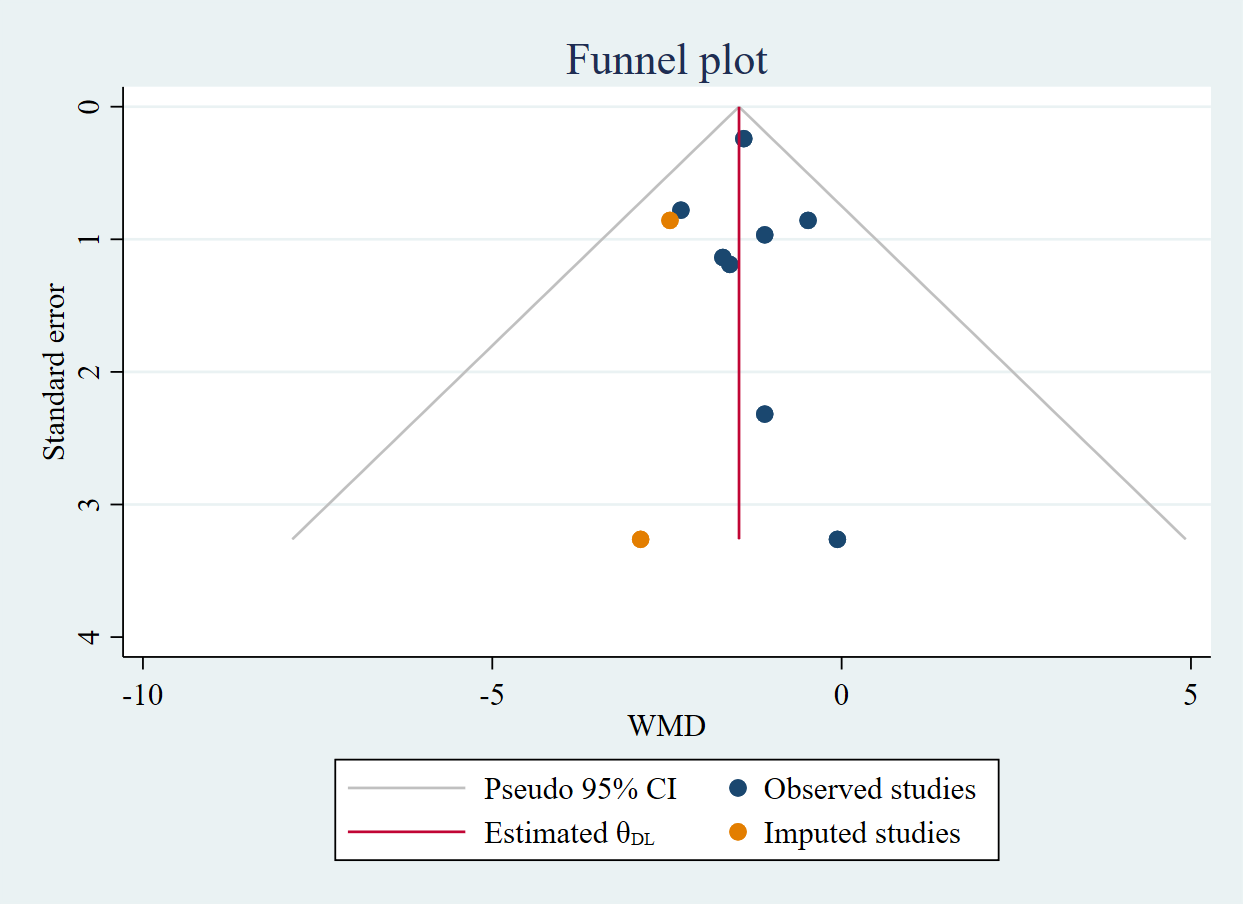


**Figure S3.** Trim-and-Fill funnel plots for weight loss.
