# Supplementary material for: Time-restricted eating with calorie restriction on weight loss and cardiometabolic risk: a systematic review and meta-analysis
Source: Eur J Clin Nutr. 2023 Jul 24;77(11):1014–25. doi: 10.1038/s41430-023-01311-w (PMC10630127; doi:10.1038/s41430-023-01311-w)
Supplement: Supplementary file 2 — supplemental tables [file 41430_2023_1311_MOESM2_ESM.docx]

**Supplementary table**

Table S1. The result of the GRADE assessment.

| **Quality assessment** | | | | | | | **No of patients** | | **Effect** | **Quality** | **Importance** |  |
| --- | --- | --- | --- | --- | --- | --- | --- | --- | --- | --- | --- | --- |
|  |  |  |  |  |  |  |  |  |  |  |  |  |
| **No of studies** | **Design** | **Risk of bias** | **Inconsistency** | **Indirectness** | **Imprecision** | **Other considerations** | **TRE+CR** | **CR** | **Absolute(95% CI)** |  |  |  |
| **weight loss** | | | | | | | | | | | |  |
| 8 | randomised trials | serious | no serious inconsistency | serious | no serious imprecision | none | 298 | 281 | MD -1.40 kg  (-1.81; -1.00) | ⊕⊕OO LOW | CRITICAL |  |
| **fat mass** | | | | | | | | | | | |  |
| 5 | randomised trials | serious | no serious inconsistency | serious | serious | none | 192 | 184 | MD -0.73 kg  (-1.39; -0.07) | ⊕OOO VERY LOW | CRITICAL |  |
| **waist circumference** | | | | | | | | | | | |  |
| 6 | randomised trials | serious | serious | serious | no serious imprecision | none | 226 | 215 | MD -1.87 cm  (-3.47; -0.26) | ⊕OOO VERY LOW | CRITICAL |  |
| **SBP** | | | | | | | | | | | |  |
| 4 | randomised trials | serious | no serious inconsistency | serious | no serious imprecision | none | 175 | 175 | MD -1.55 mmHg  (-4.09; -0.99) | ⊕⊕OO LOW | CRITICAL |  |
| **DBP** | | | | | | | | | | | |  |
| 4 | randomised trials | serious | serious | serious | no serious imprecision | none | 175 | 175 | MD -2.88 mmHg  (-6.00; 0.24) | ⊕OOO VERY LOW | CRITICAL |  |
| **glucose** | | | | | | | | | | | |  |
| 6 | randomised trials | serious | no serious inconsistency | serious | no serious imprecision | none | 234 | 227 | MD -1.67 mg/dL  (-4.69; 1.35) | ⊕⊕OO LOW | IMPORTANT |  |
| **insulin** | | | | | | | | | | | |  |
| 4 | randomised trials | serious | no serious inconsistency | serious | serious | none | 126 | 118 | MD 0.24 μIU/dL  (-1.95; 2.43) | ⊕OOO VERY LOW | IMPORTANT |  |
| **HOMA-IR** | | | | | | | | | | | |  |
| 4 | randomised trials | serious | serious | serious | serious | none | 168 | 161 | MD 0.09  (-0.85; 1.03) | ⊕OOO VERY LOW | IMPORTANT |  |
| **HOMA-β** | | | | | | | | | | | |  |
| 2 | randomised trials | serious | no serious inconsistency | serious | serious | none | 69 | 58 | MD -31.70  (-81.99; 26.59) | ⊕OOO VERY LOW | IMPORTANT |  |
| **TC** | | | | | | | | | | | |  |
| 6 | randomised trials | serious | no serious inconsistency | serious | no serious imprecision | none | 228 | 221 | MD 1.02 mg/dL  (-2.72; 4.75) | ⊕⊕OO LOW | CRITICAL |  |
| **TG** | | | | | | | | | | | |  |
| 6 | randomised trials | serious | no serious inconsistency | serious | no serious imprecision | none | 228 | 221 | MD 4.17 mg/dL  (-4.43; 12.77) | ⊕⊕OO LOW | CRITICAL |  |
| **LDL** | | | | | | | | | | | |  |
| 6 | randomised trials | serious | no serious inconsistency | serious | no serious imprecision | none | 228 | 221 | MD 1.39 mg/dL  (-1.55; 4.33) | ⊕⊕OO LOW | CRITICAL |  |

TRE: Time-restricted eating; CR: calorie restriction; MD: mean difference.
