# Supplementary material for: Time-restricted eating with calorie restriction on weight loss and cardiometabolic risk: a systematic review and meta-analysis
Source: Eur J Clin Nutr. 2023 Jul 24;77(11):1014–25. doi: 10.1038/s41430-023-01311-w (PMC10630127; doi:10.1038/s41430-023-01311-w)
Supplement: Supplementary file 4 — search terms [file 41430_2023_1311_MOESM4_ESM.docx]

Appendix S1. Search terms

(time restricted eating or time restricted feeding or time restricted fasting or time restricted diet) and (blood pressure or diastolic pressure or systolic pressure or diastolic blood pressure or systolic blood pressure or glucose or insulin or homeostatic model assessment for insulin resistance or HOMA-IR or HOMA-β or cholesterol or triglyceride* or Triacylglycerol* or plasma lipid or weight loss* or weight reduction*)

Pubmed 2022-10-18 314

(((("Time restricted feeding"[Title/Abstract]) OR ("time restricted diet"[Title/Abstract])) OR ("time restricted fasting"[Title/Abstract])) OR ("time restricted eating"[Title/Abstract])) AND ((((((((((((((((((((Blood Pressure[MeSH Terms]) OR ("blood pressure")) OR ("diastolic pressure")) OR ("systolic pressure")) OR (diastolic blood pressure)) OR ("systolic blood pressure")) OR ("glucose")) OR (insulin)) OR ("homeostatic model assessment for insulin resistance")) OR ("HOMA-IR")) OR ("HOMA-β")) ) OR (cholesterol[MeSH Terms])) OR (cholesterol)) OR (triglycerides[MeSH Terms])) OR (triglycerides)) OR (Triacylglycerols)) OR (Triacylglycerol)) OR (Triglyceride) OR ("plasma lipid")) OR (((((Weight Loss[MeSH Terms]) OR ("Weight Loss")) OR ("Weight Losses")) OR ("Weight Reduction")) OR ("Weight Reductions")))

Cochrane library 2022-10-18 921

#1 (Time restricted feeding):ti,ab,kw

#2 (time restricted diet):ti,ab,kw

#3 (time restricted fasting):ti,ab,kw

#4 (time restricted eating):ti,ab,kw

#5 #1 or #2 or #3 or #4

#6 MeSH descriptor: [Blood Pressure] explode all trees

#7 (Blood Pressure)

#8 (diastolic pressure)

#9 (systolic pressure)

#10 (diastolic blood pressure)

#11 (systolic blood pressure)

#12 (glucose)

#13 (insulin)

#14 (homeostatic model assessment for insulin resistance)

#15 (HOMA-IR)

#16 (homeostatic model assessment for β-cell function)

#17 (HOMA-β)

#18 MeSH descriptor: [Cholesterol] explode all trees

#19 (cholesterol)

#20 MeSH descriptor: [Triglycerides] explode all trees

#21 (triglycerides)

#22 (Triacylglycerols)

#23 (Triacylglycerol)

#24 (Triglyceride)

#25 (plasma lipid)

#26 MeSH descriptor: [Weight Loss] explode all trees

#27 (Weight Loss)

#28 (Weight Losss)

#29 (Weight Reduction)

#30 (Weight Reductions)

#31 #6 or #7 or #8 or #9 or #10 or #11 or #11 or #12 or #13 or #14 or #15 or #16 or #17 or #18 or #19 or #20 or #21 or #22 or #23 or #24 or #25 or #26 or #27 or #28 or #29 or #30

#32 #5 and #31

Embase 2022-10-18 374

#8 #1 AND #7

#7 #2 OR #3 OR #4 OR #5 OR #6

#6 'weight loss'/exp OR (weight AND losss) OR (weight AND reduction) OR (weight AND reductions)

#5 'cholesterol'/exp OR 'triglycerides'/exp OR triglycerides OR triacylglycerols OR triacylglycerol OR triglyceride OR (plasma AND lipid)

#4 homeostatic AND model AND assessment AND for AND insulin AND resistance OR 'homa ir' OR (homeostatic AND model AND assessment AND for AND 'β cell' AND function) OR 'homa β'

#3 glucos OR insulin

#2 'blood pressure'/exp OR (diastolic AND pressure) OR (systolic AND pressure) OR (diastolic AND blood AND pressure) OR (systolic AND blood AND pressure)

#1 'time restricted feeding':ti,ab,kw OR 'time restricted diet':ti,ab,kw OR 'time restricted fasting':ti,ab,kw OR 'time restricted eating':ti,ab,kw
